# *catena*-Poly[(dichloridozinc)-μ-4,4′-bis­[(1*H*-imidazol-1-yl)meth­yl]biphenyl-κ^2^ *N* ^3^:*N* ^3′^]

**DOI:** 10.1107/S1600536812014043

**Published:** 2012-04-13

**Authors:** Cheng Wang, Bo Wen, Zhi-Yao Sun, Peng-Fei Yan, Jin-Sheng Gao

**Affiliations:** a Key Laboratory of Functional Inorganic Material Chemistry, Ministry of Education, Heilongjiang University, Harbin 150080, People’s Republic of China; b Engineering Research Center of Pesticide of Heilongjiang University, Heilongjiang University, Harbin 150050, People’s Republic of China

## Abstract

In the title compound, [ZnCl_2_(C_20_H_18_N_4_)]_*n*_, the Zn^II^ ion lies on a twofold rotation axis and is four-coordinated in a tetra­hedral geometry defined by two Cl anions and two N atoms from two 4,4′-bis­[(imidazol-1-yl)meth­yl]biphenyl ligands. The mid-point of the ligand is located on an inversion center, and shows a *trans* conformation. The ligands link the Zn^II^ ions, forming a chain structure along [10-1].

## Related literature

For the synthesis of the ligand, see: Zhu *et al.* (2002 ▶).
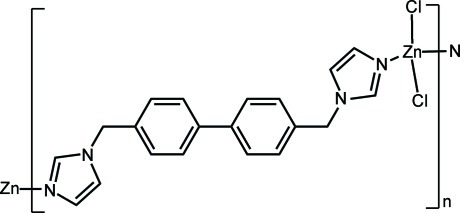


## Experimental

### 

#### Crystal data

[ZnCl_2_(C_20_H_18_N_4_)]
*M* *_r_* = 450.67
Monoclinic, 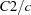

*a* = 22.837 (5) Å
*b* = 5.9004 (12) Å
*c* = 16.012 (3) Å
β = 117.08 (3)°
*V* = 1921.0 (9) Å^3^
*Z* = 4
Mo *K*α radiation
μ = 1.57 mm^−1^
*T* = 293 K
0.36 × 0.20 × 0.18 mm

#### Data collection

Rigaku R-AXIS RAPID diffractometer
Absorption correction: multi-scan (*ABSCOR*; Higashi, 1995 ▶) *T*_min_ = 0.601, *T*_max_ = 0.765
8827 measured reflections
2204 independent reflections
1890 reflections with *I* > 2σ(*I*)
*R*_int_ = 0.027

#### Refinement

*R*[*F* ^2^ > 2σ(*F* ^2^)] = 0.031
*wR*(*F* ^2^) = 0.083
*S* = 1.07
2204 reflections
123 parameters
H-atom parameters constrained
Δρ_max_ = 0.32 e Å^−3^
Δρ_min_ = −0.24 e Å^−3^

### 

Data collection: *RAPID-AUTO* (Rigaku, 1998 ▶); cell refinement: *RAPID-AUTO*; data reduction: *CrystalClear* (Rigaku/MSC, 2002 ▶); program(s) used to solve structure: *SHELXS97* (Sheldrick, 2008 ▶); program(s) used to refine structure: *SHELXL97* (Sheldrick, 2008 ▶); molecular graphics: *SHELXTL* (Sheldrick, 2008 ▶); software used to prepare material for publication: *SHELXTL*.

## Supplementary Material

Crystal structure: contains datablock(s) I, global. DOI: 10.1107/S1600536812014043/hy2529sup1.cif

Structure factors: contains datablock(s) I. DOI: 10.1107/S1600536812014043/hy2529Isup2.hkl

Additional supplementary materials:  crystallographic information; 3D view; checkCIF report

## Figures and Tables

**Table 1:** Selected bond lengths (Å)

Zn1—Cl1	2.2349 (9)
Zn1—N1	2.0224 (16)

**Fig. 1.**
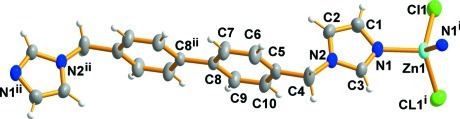
The asymmetric unit of the title compound, showing displacement ellipsoids at the 50% probability level. [Symmetry codes: (i) -x, y, 1/2-z; (ii) 1/2-x, -1/2-y, -z.]

**Fig. 2.**
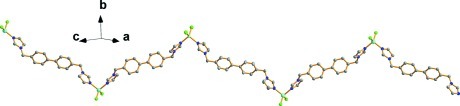
A partial packing view, showing the chain structure along [1 0 1].

## supplementary crystallographic information

### Comment

N-containing ligands with an arene center have been widely used as building blocks for constructing inorganic-organic supramolecular architectures. Herein, we report the title compound constructed from 4,4'-(dimethylenebiphenyl)diimidazole and ZnCl_2_.

In the title compound, the Zn^II^ ion lies on a twofold rotation axis and is four-coordinated in a tetrahedral environment defined by two Cl anions and two N atoms from two ligands (Fig. 1, Table 1). The mid-point of the ligand is located in an inversion center. The ligands showing a *trans* conformation link the Zn^II^ ions into a chain structure along [1 0 1] (Fig. 2).

### Experimental

The 4,4'-(dimethylenebiphenyl)diimidazol ligand was synthesized followed the reference method (Zhu *et al.*, 2002). ZnCl_2_ (0.140 g, 1 mmol) and ligand (0.32 g, 1 mmol) were dissolved in a mixed solution of 4 ml ethanol and 4 ml water. After stirring the suspension was sealed in a 18 ml Teflon-lined autoclave and heated at 140°C for 5 days. After slow cooling to room temperature, colorless block crystals were filtered and washed with distilled water (yield: 35% based on Zn).

### Refinement

H atoms bound to C atoms were placed in calculated positions and treated as riding atoms, with C—H = 0.93 (aromatic) and 0.97 (methylene) Å and with *U*_iso_(H) = 1.2*U*_eq_(C).

### Crystal data

**Table:**

[ZnCl_2_(C_20_H_18_N_4_)]	*F*(000) = 920
*M**_r_* = 450.67	*D*_x_ = 1.558 Mg m^−^^3^
Monoclinic, *C*2/*c*	Mo *K*α radiation, λ = 0.71073 Å
Hall symbol: -C 2yc	Cell parameters from 7615 reflections
*a* = 22.837 (5) Å	θ = 3.6–27.5°
*b* = 5.9004 (12) Å	µ = 1.57 mm^−^^1^
*c* = 16.012 (3) Å	*T* = 293 K
β = 117.08 (3)°	Block, colorless
*V* = 1921.0 (9) Å^3^	0.36 × 0.20 × 0.18 mm
*Z* = 4	

### Data collection

**Table:**

Rigaku R-AXIS RAPID diffractometer	2204 independent reflections
Radiation source: fine-focus sealed tube	1890 reflections with *I* > 2σ(*I*)
Graphite monochromator	*R*_int_ = 0.027
ω scan	θ_max_ = 27.5°, θ_min_ = 3.6°
Absorption correction: multi-scan (*ABSCOR*; Higashi, 1995)	*h* = −29→26
*T*_min_ = 0.601, *T*_max_ = 0.765	*k* = −7→7
8827 measured reflections	*l* = −20→20

### Refinement

**Table:**

Refinement on *F*^2^	Primary atom site location: structure-invariant direct methods
Least-squares matrix: full	Secondary atom site location: difference Fourier map
*R*[*F*^2^ > 2σ(*F*^2^)] = 0.031	Hydrogen site location: inferred from neighbouring sites
*wR*(*F*^2^) = 0.083	H-atom parameters constrained
*S* = 1.07	*w* = 1/[σ^2^(*F*_o_^2^) + (0.0483*P*)^2^ + 0.6927*P*] where *P* = (*F*_o_^2^ + 2*F*_c_^2^)/3
2204 reflections	(Δ/σ)_max_ < 0.001
123 parameters	Δρ_max_ = 0.32 e Å^−^^3^
0 restraints	Δρ_min_ = −0.24 e Å^−^^3^

### Special details

**Table:**

Geometry. All esds (except the esd in the dihedral angle between two l.s. planes) are estimated using the full covariance matrix. The cell esds are taken into account individually in the estimation of esds in distances, angles and torsion angles; correlations between esds in cell parameters are only used when they are defined by crystal symmetry. An approximate (isotropic) treatment of cell esds is used for estimating esds involving l.s. planes.
Refinement. Refinement of F^2^ against ALL reflections. The weighted R-factor wR and goodness of fit S are based on F^2^, conventional R-factors R are based on F, with F set to zero for negative F^2^. The threshold expression of F^2^ > 2sigma(F^2^) is used only for calculating R-factors(gt) etc. and is not relevant to the choice of reflections for refinement. R-factors based on F^2^ are statistically about twice as large as those based on F, and R- factors based on ALL data will be even larger.

### Fractional atomic coordinates and isotropic or equivalent isotropic displacement parameters (Å^2^)

**Table:**

	*x*	*y*	*z*	*U*_iso_*/*U*_eq_	
C1	0.02165 (11)	0.3388 (4)	0.10470 (15)	0.0471 (5)	
H1	−0.0130	0.4089	0.0544	0.057*	
C2	0.05856 (10)	0.1682 (4)	0.09746 (15)	0.0456 (5)	
H2	0.0543	0.1002	0.0426	0.055*	
C3	0.09174 (10)	0.2535 (3)	0.24453 (14)	0.0392 (4)	
H3	0.1152	0.2508	0.3096	0.047*	
C4	0.15012 (12)	−0.0734 (4)	0.21863 (17)	0.0494 (5)	
H4A	0.1281	−0.2080	0.2249	0.059*	
H4B	0.1856	−0.0379	0.2800	0.059*	
C5	0.17867 (10)	−0.1230 (3)	0.15169 (14)	0.0389 (4)	
C6	0.16437 (12)	−0.3221 (4)	0.10166 (18)	0.0502 (5)	
H6	0.1358	−0.4253	0.1077	0.060*	
C7	0.19175 (12)	−0.3717 (4)	0.04240 (18)	0.0499 (5)	
H7	0.1810	−0.5075	0.0093	0.060*	
C8	0.23486 (8)	−0.2230 (3)	0.03141 (12)	0.0317 (4)	
C9	0.24832 (10)	−0.0205 (4)	0.08169 (15)	0.0440 (5)	
H9	0.2766	0.0840	0.0756	0.053*	
C10	0.22065 (11)	0.0283 (4)	0.14005 (16)	0.0482 (5)	
H10	0.2303	0.1653	0.1722	0.058*	
Cl1	−0.07645 (3)	0.79888 (10)	0.12779 (4)	0.05314 (16)	
N1	0.04281 (8)	0.3932 (3)	0.19687 (12)	0.0385 (4)	
N2	0.10332 (8)	0.1157 (3)	0.18714 (12)	0.0366 (3)	
Zn1	0.0000	0.61108 (5)	0.2500	0.03718 (12)	

### Atomic displacement parameters (Å^2^)

**Table:**

	*U*^11^	*U*^22^	*U*^33^	*U*^12^	*U*^13^	*U*^23^
C1	0.0445 (10)	0.0614 (13)	0.0393 (11)	0.0076 (10)	0.0223 (9)	0.0016 (9)
C2	0.0460 (10)	0.0579 (12)	0.0390 (10)	−0.0028 (10)	0.0247 (9)	−0.0125 (9)
C3	0.0477 (10)	0.0418 (10)	0.0378 (10)	0.0014 (9)	0.0280 (8)	−0.0045 (8)
C4	0.0707 (14)	0.0445 (11)	0.0551 (13)	0.0143 (10)	0.0479 (11)	0.0067 (9)
C5	0.0459 (10)	0.0392 (10)	0.0435 (10)	0.0063 (8)	0.0307 (9)	0.0010 (8)
C6	0.0615 (13)	0.0434 (10)	0.0690 (15)	−0.0130 (10)	0.0500 (12)	−0.0104 (10)
C7	0.0638 (13)	0.0412 (11)	0.0669 (14)	−0.0164 (10)	0.0491 (12)	−0.0197 (10)
C8	0.0333 (8)	0.0326 (9)	0.0358 (9)	−0.0002 (7)	0.0214 (7)	−0.0040 (7)
C9	0.0530 (11)	0.0374 (10)	0.0573 (13)	−0.0125 (9)	0.0388 (10)	−0.0123 (9)
C10	0.0639 (13)	0.0397 (10)	0.0576 (13)	−0.0078 (10)	0.0422 (11)	−0.0151 (10)
Cl1	0.0633 (3)	0.0527 (3)	0.0479 (3)	0.0107 (3)	0.0292 (3)	0.0057 (2)
N1	0.0449 (9)	0.0399 (8)	0.0424 (9)	0.0015 (7)	0.0302 (7)	−0.0015 (7)
N2	0.0424 (8)	0.0382 (8)	0.0400 (8)	0.0005 (7)	0.0281 (7)	−0.0036 (6)
Zn1	0.0457 (2)	0.03405 (18)	0.0461 (2)	0.000	0.03332 (15)	0.000

### Geometric parameters (Å, º)

**Table:**

C1—C2	1.352 (3)	C5—C10	1.383 (3)
C1—N1	1.366 (3)	C6—C7	1.385 (3)
C1—H1	0.9300	C6—H6	0.9300
C2—N2	1.366 (3)	C7—C8	1.389 (3)
C2—H2	0.9300	C7—H7	0.9300
C3—N1	1.317 (3)	C8—C9	1.395 (3)
C3—N2	1.340 (2)	C8—C8^i^	1.491 (3)
C3—H3	0.9300	C9—C10	1.376 (3)
C4—N2	1.467 (3)	C9—H9	0.9300
C4—C5	1.515 (3)	C10—H10	0.9300
C4—H4A	0.9700	Zn1—Cl1	2.2349 (9)
C4—H4B	0.9700	Zn1—N1	2.0224 (16)
C5—C6	1.375 (3)		
			
C2—C1—N1	109.87 (19)	C8—C7—H7	119.3
C2—C1—H1	125.1	C7—C8—C9	116.80 (16)
N1—C1—H1	125.1	C7—C8—C8^i^	121.6 (2)
C1—C2—N2	106.02 (18)	C9—C8—C8^i^	121.6 (2)
C1—C2—H2	127.0	C10—C9—C8	121.50 (18)
N2—C2—H2	127.0	C10—C9—H9	119.3
N1—C3—N2	111.25 (18)	C8—C9—H9	119.3
N1—C3—H3	124.4	C9—C10—C5	121.18 (19)
N2—C3—H3	124.4	C9—C10—H10	119.4
N2—C4—C5	112.62 (17)	C5—C10—H10	119.4
N2—C4—H4A	109.1	C3—N1—C1	105.60 (16)
C5—C4—H4A	109.1	C3—N1—Zn1	126.82 (14)
N2—C4—H4B	109.1	C1—N1—Zn1	127.08 (14)
C5—C4—H4B	109.1	C3—N2—C2	107.25 (16)
H4A—C4—H4B	107.8	C3—N2—C4	124.48 (18)
C6—C5—C10	117.91 (18)	C2—N2—C4	127.82 (17)
C6—C5—C4	120.83 (18)	N1—Zn1—N1^ii^	101.05 (9)
C10—C5—C4	121.25 (18)	N1—Zn1—Cl1^ii^	110.42 (5)
C5—C6—C7	121.24 (19)	N1^ii^—Zn1—Cl1^ii^	106.35 (5)
C5—C6—H6	119.4	N1—Zn1—Cl1	106.35 (5)
C7—C6—H6	119.4	N1^ii^—Zn1—Cl1	110.42 (5)
C6—C7—C8	121.37 (18)	Cl1^ii^—Zn1—Cl1	120.56 (4)
C6—C7—H7	119.3		

Symmetry codes: (i) −*x*+1/2, −*y*−1/2, −*z*; (ii) −*x*, *y*, −*z*+1/2.
